# Exercise and Physical Activity as Modifiable Risk Factors for Benign Prostatic Hyperplasia: An Update

**DOI:** 10.1007/s11934-026-01346-z

**Published:** 2026-07-21

**Authors:** Frederico Branco, Sílvia Rocha-Rodrigues, María J. Ribal, Ricardo Ribeiro

**Affiliations:** 1 Urology Department, Prelada Hospital, Rua de Sarmento de Beires, Porto, Portugal; 2 i3S - Institute of Investigation and Innovation in Health, University of Porto, Porto, Portugal; 3 Escola Superior Desporto e Lazer, Instituto Politécnico de Viana do Castelo, Viana do Castelo, Portugal; 4 Sport Physical Activity and Health Research & Innovation Center (SPRINT), Viana do Castelo, Portugal; 5 Uro-Oncology Unit, Hospital Clínic de Barcelona-University of Barcelona, Barcelona, Spain; 6 Clinic of Genetics and Pathology, Santo António University Hospital Centre, Porto, Portugal; 7 School of Medicine and Biomedical Sciences, University Fernando Pessoa, Porto, Portugal; 8 ICBAS - School of Medicine and Biomedical Sciences, University of Porto, Porto, Portugal

**Keywords:** Benign prostatic hyperplasia, Exercise, Lower urinary tract symptoms, Physical activity

## Abstract

**Purpose of Review:**

Benign prostatic hyperplasia (BPH) is a highly prevalent urological condition in aging men, contributing to lower urinary tract symptoms (LUTS) and reducing the quality of life. The increasing burden of BPH underscores the need for preventive strategies targeting modifiable risk factors. This review examines the evidence on the association between physical activity (PA), exercise, and BPH risk, aiming to determine whether these two forms of activity exert different effects on BPH pathogenesis.

**Recent Findings:**

A literature search was conducted in multiple databases to identify human studies evaluating the association between PA or exercise and the risk of developing BPH and/or LUTS. A total of 19 studies were included: 17 observational studies, one meta-analysis, and one two-sample Mendelian randomization study. Among the six studies evaluating exercise, four reported a protective effect against BPH development, while two found no significant association. Moderate-intensity exercise appeared to confer greater protection than high-intensity exercise. Of the 14 studies assessing PA, seven reported a protective effect, with most indicating that high-intensity PA provided the greatest benefit. Two studies yielded mixed results, while five found no significant association between PA and BPH risk.

**Summary:**

Although moderate-intensity exercise and moderate-to-high PA appear to reduce BPH risk, evidence remains inconsistent. Future research should prioritize randomized controlled trials with standardized protocols, clearly defined outcomes, and objective assessment of dose–response effects to elucidate underlying mechanisms. Such approaches are critical to clarify the role of exercise and PA in BPH prevention and to inform clinical and public health recommendations.

## Introduction

Benign prostatic hyperplasia (BPH) is one of the most prevalent urological conditions in aging men and is characterized by a non-malignant enlargement of the prostate gland [1, 2]. Despite being incompletely understood, it is believed that the pathophysiology of BPH is driven by unregulated hyperplastic growth of epithelial and fibromuscular tissues within the transition zone and periurethral region [3]. This results in compression of the urethra and obstruction of bladder outflow, potentially causing lower urinary tract symptoms (LUTS), urinary retention, or infections stemming from inadequate bladder emptying [3]. If left untreated, BPH can result in chronic high-pressure urinary retention, posing a life-threatening risk and causing long-term or irreversible changes to the detrusor muscle [4].

The prevalence of histological BPH identified in autopsy studies rises significantly with age, reaching 90% in men aged 81–90 years [5]. Additionally, LUTS are associated with substantial morbidity and have been shown to exert a greater impact on anxiety and depression than other chronic conditions such as diabetes, gout, and hypertension [5–7]. The burden of BPH/LUTS is expected to rise significantly, impacting both patients and the healthcare system. This increase is driven primarily by global population aging, with individuals over 65 years projected to grow from 9.06% (680 million) in 2018 to 17% (1.6 billion) within the next 30 years [6, 7].

In addition to advancing age, risk factors for BPH include family history [8, 9], hormonal imbalances [10–12], African American ethnicity [13–15], obesity [16–19], metabolic syndrome [20], type 2 diabetes mellitus [21–23], red meat intake [24, 25] and physical inactivity [26]. Higher levels of physical activity (PA) and exercise have been associated with lower risk of BPH and LUTS, though findings remain inconsistent, suggesting that the effect may vary depending on study designs, PA and exercise intensity and frequency, and methodological approaches [26–28]. Given this variability, it is important to distinguish between PA and exercise, as the terms are often used interchangeably in medical literature despite having distinct definitions [29]. PA includes any movement produced by skeletal muscles that expends energy, encompassing exercise, leisure activities, active transportation, and occupational or domestic tasks, while exercise is a structured, repetitive subset of PA designed to improve or maintain physical fitness [29].

Therefore, considering the significant impact of BPH/LUTS on men’s health and quality of life [30], and the increasing burden in the economic and healthcare systems [31], there is a clear need to develop effective preventive measures by identifying and addressing modifiable risk factors that can significantly reduce disease susceptibility and progression. This narrative review aims to summarize and critically discuss current evidence on the relationship between PA and exercise and the development and progression of BPH and LUTS.

## Methods

The literature search included peer-reviewed articles from multiple databases, including MEDLINE (PubMed), Google Scholar, the Cochrane Library Central Register of Controlled Trials, Embase, and ClinicalTrials.gov, covering the period from 1990 to May 19th 2024. In addition, grey literature sources were consulted, and the reference lists of the retrieved articles were reviewed to identify any further relevant publications. Search terms related to PA and exercise (e.g., physical activity, exercise, training, sports, fitness) were combined with terms for BPH (e.g., benign prostatic hyperplasia, BPH) using database filters to retrieve relevant results based on article titles and abstracts. Inclusion criteria comprised observational and interventional studies in humans, published in English, that examined structured exercise and PA, including leisure-time sports, such as walking, climbing, tennis, and swimming. Studies investigating LUTS associated with BPH were also included due to their established link with its pathophysiology. BPH cases were identified using one or more of the following outcomes: non-cancerous prostate surgery, prostate volume or weight, urine flow rate, medical treatment, self-reported physician diagnosis, clinical markers (e.g., PSA levels) or classification according to the N40 code of the International Classification of Diseases, 10th Revision (ICD-10), and the 600 code in the 8th and 9th revisions (ICD-8 and ICD-9). The outcomes for LUTS included the American Urological Association Symptom Index (AUA-SI) or International Prostate Symptom Score (IPSS) questionnaires, difficulty in urination, as well as presence of nocturia. Exclusion criteria comprised articles focusing on occupational PA related to work or job activities, domestic tasks, and mind-body exercises such as Tai Chi. The literature search resulted in 19 articles, most of which were observational and relied on self-reported PA or exercise.

## Results

**Fig. 1:**
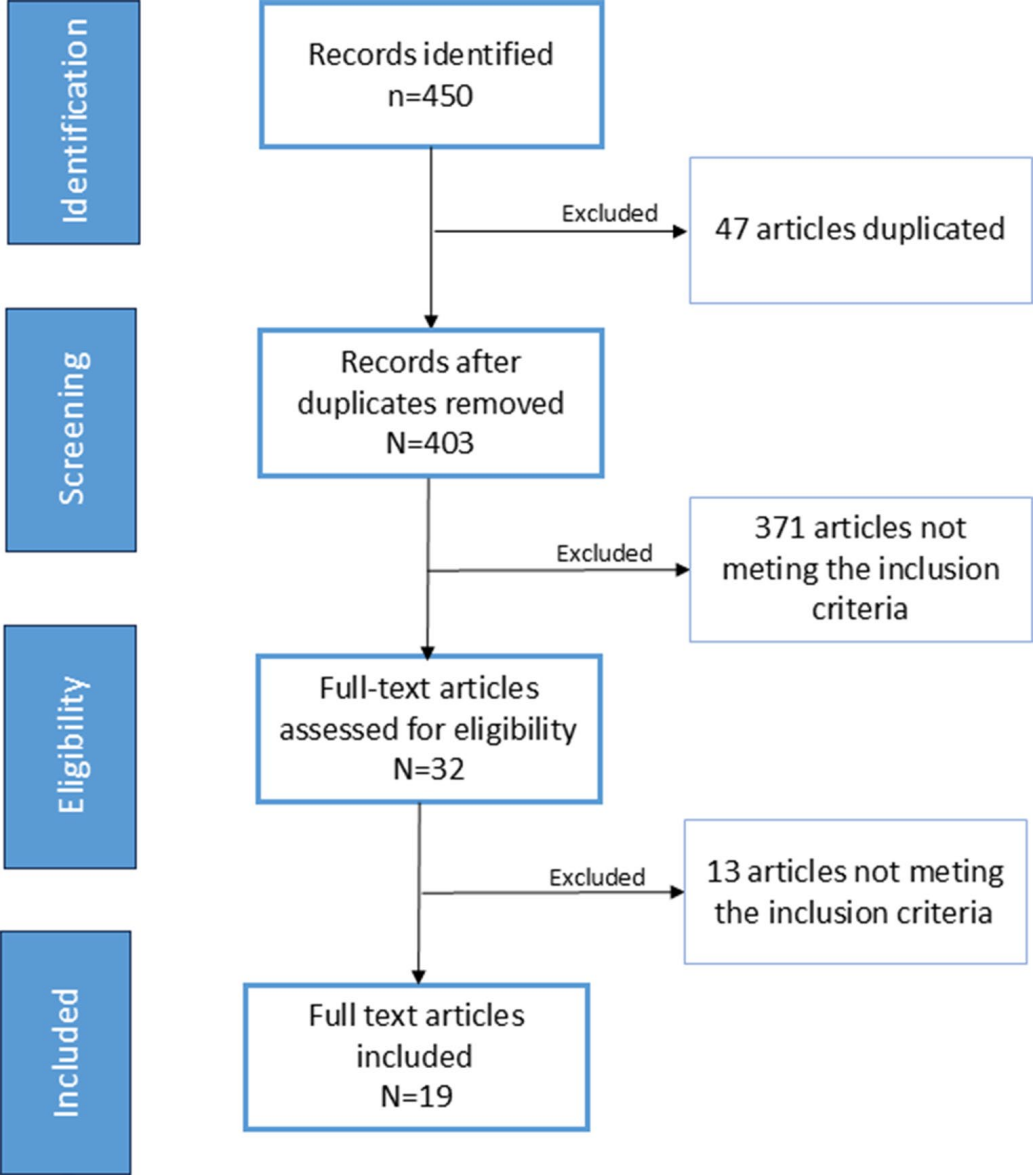
Flow diagram of the literature search and study selection process

The literature search investigating the association between PA or exercise and the risk of developing BPH initially identified 450 records. After removing 47 duplicates, 403 abstracts were screened based on their titles and abstracts. Of these, 32 studies were deemed relevant and selected for full-text review to assess their eligibility. Following a detailed evaluation, 13 studies were excluded for not meeting the predefined inclusion criteria, resulting in a final selection of 19 studies. The selection and review strategy are outlined in the flow diagram presented in Fig. 1.

Of the 19 studies included in this review, six studies assessing the relationship between exercise and BPH risk are presented in Table 1, while 14 studies investigating the association between PA and BPH risk are summarized in Table 2. The study of Lee et al. (2014) [32] is presented in both tables, as it evaluated both exercise frequency (Table 1) and PA during leisure time (Table 2).

**Table 1:** Summary of the literature evaluating the association between exercise and risk of developing benign prostatic hyperplasia

Reference	Study Design	Sample Size	Age/ Follow-up or Age-associated exposures	Endpoint/Definition	Exercise	Findings
Gann et al., 1995	Case control	*N* = 640 *n* = 320 BPH cases *n* = 320 controls	40–84 years 9- year follow-up	BPH Surgery	Exercise frequency: 1–3 times/month, 1, 2–4 or + 5 times/week	Rare/never: OR 1.0 (reference) 1–3 times/month: OR 0.24 (95% CI, 0.11–0.55) 1 time/week: OR 0.22 (95% CI, 0.10–0.50) 2–4 times/week: OR 0.46 (95% CI, 0.24–0.89) + 5 times/week: OR 0.70 (95% CI, 0.32–1.51)
Hong et al., 2006	Cross-sectional	*N* = 641	50–79 years	BPH IPSS (> 8) prostate weight (> 25 g), and maximal flow rate of < 15 ml/s	Exercise frequency: <2, 3–5 h/week, nearly everyday	< 2 h/week: OR 1.0 (reference) 3–5 h/week: OR 0.48 (95% CI, 0.23–1.00) Nearly everyday: OR 1.73 (95% CI, 0.96–3.10)
Williams et al., 2008	Cohort study	*N* = 28 612 runners *n* = 1899 BPH cases	7.7- year follow-up	BPH Physician diagnosed (not specified)	Distance run/week and performance (10 km)	Compared with ≤ 16 km/week (guideline levels), running 16–32, 32–48, 48–64, and > 64 km/week reduced BPH risk by 6%, 12%, 24%, and 33%, respectively.
Lee et al., 2014	Cross-sectional	*N* = 582 *N* = 114 BPH cases	≥ 40 years	BPH/LUTS Evaluation based on IPSS, digital rectal examination, PSA screening, and prostate size BPH was defined as ≥ 25 mL of prostate volume and an IPSS score of ≥ 8	Regular exercise (absence or presence of sufficient amount of body sweat); exercise frequency (1–4 or ≥ 5/ week); exercise time (< 60 min/once or ≥ 60 min/once); sedentary time (4.50–7-00 or >7 h/day); non-sedentary time (1.79–3.60 or > 3.60 h/day; leisure time (0.06 − 0.054 or > 0.54 h/day) and leisure time PA (≥ 22 MET h/week)	Regular exercise was reported as a risk factor for BPH (OR 1.12; 95% CI, 0.66–1.88). Exercising ≥ 5 times per week appeared to have a higher risk (OR 1.26; 95% CI, 0.68–2.33), although no significant linear trend was observed (P for trend = 0.52). Exercising for at least 60 min was also suggested as a potential risk factor (OR 1.05; 95% CI, 0.60–1.97), but no overall trend was detected (P for trend = 0.78).”
Nagakura et al., 2022	Cross-sectional	*N* = 29 million	40–74 years	BPH Two BPH drugs: uroselective α1 receptor blocker and 5α-reductase inhibitor/ dutasteride	Exercise habits: sweaty exercise (30 min or more per session and 2 days or more/week) for over a year (%Yes/No).	Inverse correlation between exercise habits and uroselective α1 receptor blocker (*r* = −0.5696, 95% CI: −0.7364 ~ −0.3377; *p* < 0.01 by t-test)
Jia et al., 2024	Two-sample MR study	*N* = 64 949	NA	BPH According to ICD-10 code N40; ICD-9 code 600; ICD-8 code 600	Exercise levels (light, moderate and vigorous)	No association between exercise and BPH

*BPH* benign prostatic hyperplasia, *CI * confidence interval, *g *grams, *h* hour, *ICD-8* International Classification of Diseases, 8th Revision, *ICD-9* International Classification of Diseases, 9th Revision, *ICD-10* International Classification of Diseases, 10th Revision, *IPSS* International Prostate Symptom Score, *km* kilometers, *LUTS* lower urinary tract symptoms, *MET * metabolic equivalent of task, *mL* milliliters, *MR* Mendelian randomization, *NA * not available, *OR* odds ratio, *PSA* prostate-specific antigen, *r* correlation coefficient

**Table 2:** Summary of the literature evaluating the association between physical activity and the risk of developing benign prostatic hyperplasia

Reference	Study Design	Sample Size	Age range/ Follow-up or Age-associated exposures	Endpoint/Definition	Physical Activity	Findings
Platz et al., 1998	Cohort study	*N* = 30 634 *n* = 3743 BPH cases (1890 cases of surgery for BPH, and 1853 symptomatic BPH cases)	40–75 years 8-year follow-up	BPH/LUTS Surgery, symptoms, or digital rectal examination (prostatic enlargement) Symptomatic BPH cases were stratified according to severity using the AUA-SI	Time/week engaging in activities like walking, hiking outdoors, jogging, running, bicycling, tennis, number of flights climbed, etc. Highest quintile of exercise: ≥33.8 MET-h/week Lowest quintile of exercise: 0.1 to 3 MET-h/week	Total BPH: OR 0.75 (95% CI, 0.67–0.85) Surgery for BPH: OR 0.76 (95% CI, 0.64–0.90) Symptomatic BPH: OR 0.75 (95% CI, 0.64–0.87) Men who walked 2 to 3 h/week had a 25% lower risk of total BPH. Total BPH risk: low-to-moderate intensity (OR 0.90; 95% CI, 0.86–0.93) was beneficial than high-intensity (OR 0.97; 95% CI, 0.95–1.00) PA
Lacey et al., 2001	Case control	*N* = 677 *n* = 206 BPH cases *n* = 471 controls	Ages: 20–29 years 40–49 years 50–94 years	BPH Surgery	Moderate or vigorous energy expenditure (MET-h/week) All activity, high vs. sedentary	No association between PA and BPH
Meigs et al., 2001	Cohort study	*N* = 1019	40–79 years 9-year follow-up	BPH/LUTS Surgery, difficult or frequent urination and enlarged or swollen prostate	Frequency and duration of activities in last 7 days (moderate, vigorous and heavy categories; total energy expenditure, kcal/day)	High levels of PA (top vs. bottom quartile of total energy expenditure) decreased odds of BPH (OR 0.5 (95% CI, 0.3–0.9; *P* = 0.002 for trend)
Prezioso et al., 2001	Cohort study	*N* = 1033	NA	BPH/LUTS Prostate volume and evaluation of symptoms based on IPSS	Reported PA	Compared to sedentary, subjects regularly engaged in PA showed lower: Prostate volume: 44 vs. 50 cm^3^, *p* = 0.04 IPSS: 15 vs. 13, *p* = 0.008 IPSS-QoL: 2.7 vs. 3.1, *p* = 0.007 Frequency of incomplete emptying: 73 vs. 83%, *p* = 0.02) Repeated urination: 83 vs. 91%, *p* = 0.02 Intermittence: 67 vs. 79%, *p* = 0.02 Urgency: 60 vs. 74%, *p* = 0.01
Joseph et al., 2003	Case control	*N* = 708	40–79 years	LUTS Evaluation of symptoms based on AUA-SI	Average number of h/day engaged in vigorous activities that required to work up a sweat, and weekly pattern of exercise	No association between PA and BPH
Maso et al., 2006	Case control	*N* = 2820 *n* = 1369 BPH cases *n* = 1451 controls	46–74 years Ages: 15–19 years 30–39 years 50–59 years	BPH Surgery with histological confirmation	Recreational PA (e.g., walking, cycling, gardening): number of hours of activity per week (< 2, 2–4, ≥ 5 h/week)	< 2 vs. ≥5 h/week: OR 0.5 (95% CI, 0.4–0.7) at age 15–19 OR 0.6 (95% CI, 0.5–0.8) at age 30–39 OR 0.7 (95% CI, 0.5–0.8) at age 50–59 The strongest association emerged with PA in young adulthood (~ 50% of risk reduction), but some protection was also found for high PA levels at ages 50–59 years (one-third reduction)
Rohrmann et al., 2006	Twin case–control study	*N* = 3446 1723 twin pairs	Median age: 74 years	LUTS Evaluation of moderate/severe symptoms based on IPSS	High vs. low PA score, based on the daily number of flights of stairs climbed and the daily number of blocks walked	High-moderate/severe (15+) vs. low (≤ 7) IPSS: Low PA score: OR 1 (reference)^c^ Medium PA score: OR 0.70 (95% CI, 0.40–1.22) High PA score: OR 0.60 (95% CI, 0.34–1.08)
Kristal et al., 2007	Cohort study	*N* = 5667	54–86 years 7-year follow-up	BPH Surgery or medical treatment and evaluation of symptoms by IPSS	Sedentary vs. highly active	No association between PA and BPH
Fritschi et al., 2007	Case control	*N*=869 *n* = 398 BPH cases *n* = 471 controls	40–75 years Ages: 12–18 years 19–34 years 35–49 years > 50 years	BPH Surgery	Moderate vs. vigorous PA since age 12. Moderate intensity: 3–5.9 METs Vigorous intensity: ≥6 METs	No association between PA and BPH
Safarinejad (2008)	Cross-sectional	*N* = 8466	≥ 40 3-year follow-up	BPH Prostate size, urine flow and IPSS (> 7) to evaluate symptoms	Reported PA (kcal/day)	≥ 2,000 vs. ≤ 500 kcal/day: OR 0.4 (95% CI, 0.31–1.24, *p* = 0.01)
Parsons and Kashefi 2008	Meta-analysis	*N* = 35 675	NA	BPH/LUTS	PA was stratified in: light, moderate, and vigorous categories (sedentary category was the reference)	Light PA: OR 0.70 (95% CI, 0.44–1.13, *p* = 0.14) Moderate PA: OR 0.74 (95% CI, 0.60–0.92, *p* = 0.005) Vigorous PA: OR 0.74 (95% CI, 0.59–0.92, *p* = 0.006)
Fowke et al., 2013	Cross-sectional	*N* = 405	40–93 years	LUTS/BPH Evaluation of symptoms based on AUA-SI and prostate volume	Baecke questionnaire using MET-h/day to estimate leisure-time PAEE	Higher PAEE was significantly associated with lower LUTS severity (*P* = 0.041), but not with prostate volume (*P* = 0.256)
Lee et al., 2014	Cross-sectional	*N* = 582	≥ 40 years	BPH/LUTS Evaluation based on IPSS, digital rectal examination, PSA screening, and prostate size BPH was defined as ≥ 25 mL of prostate volume and an IPSS score of ≥ 8.	Non-sedentary time (1.79–3.60 or > 3.60 h/day; leisure time (0.06 − 0.054 or > 0.54 h/day) and LTPA (≥ 22 MET-h/week)	BPH was higher among those with the highest sedentary time. Analysis of LTPA in all subjects showed that those with LTPA > 22.5, had significantly larger prostate volumes (*P* = 0.024, P for trend < 0.001). Those with lower leisure time (0.06–0.54 h/day) were at an increased risk of BPH (OR 0.95; 95% CI, 0.50–1.82) compared to the higher leisure time group (OR 0.89; 95% CI, 0.51–1.54), but this association was not statistically significant. In terms of LTPA, no statistically significant differences were observed when comparing the group with ≥ 22.5 METs with the group with < 22.5 (OR 0.94; 95% CI, 0.53–1.67)
Wolin et al., 2015	Cohort study	*N* = 28 404 (prevalent analysis) *N* = 4710 (incident analysis)	40 years	BPH/LUTS Prevalent and incident BPH-related outcomes (measured by self-report of physician diagnosis, BPH surgery, finasteride use, and clinical indicators: PSA and prostate volume) and nocturia alone	Type of vigorous activities (e.g., swimming) and their levels at 40 year of age in categories of hours per week (none, < 1, 1,2, 3, and ≥ 4 h/week). Frequency in the last year and duration of each session	No significant association between PA and most BPH-related outcomes, except for nocturia. Prevalent analysis: men reporting ≥ 1 h/week of PA had 15% (95% CI, 5%–25%) lower prevalence of severe nocturia. Incident analysis: active men (≥ 1 h/week) were 13% less likely (95% CI, 2%–22%) to report nocturia and 34% less likely (95% CI, 15%–49%) to report severe nocturia as compared with men who reported no PA.

*AUA-SI* American Urological Association Symptom Index, *BPH* benign prostatic hyperplasia, *CI* confidence interval, *h* hour, *IPSS* International Prostate Symptom Score, *kcal* kilocalories, *LTPA* leisure-time physical activity, *LUTS* lower urinary tract symptoms, *MET* metabolic equivalent of task, *OR* odds ratio, *PA* physical activity, *PAEE* physical activity energy expenditure, *PSA* prostate-specific antigen, *QoL* quality of life

## Exercise and the Risk of BPH

Table 1 provides an overview of the key characteristics of the six studies investigating the association between exercise and BPH risk, comprising one case control study [33], three cross-sectional studies [32, 34, 35], one cohort study [36] and one two-sample Mendelian randomization study [37]. Four of the six studies [33–36] demonstrated an inverse correlation between exercise frequency and BPH risk, indicating that exercise reduces the risk of developing BPH. In contrast, two studies [32, 37] did not establish a significant association between exercise and BPH risk.

Among the four studies reporting a protective effect, two found a nonlinear association between exercise frequency and BPH risk, where moderate levels of exercise were associated with a lower risk, but higher frequencies did not confer additional benefits and, in some cases, were linked to an increased risk of developing BPH [33, 34]. Gann et al. (1995) reported that men engaging in 1–3 exercise sessions per month or once per week had the lowest BPH risk, while those exercising ≥ 5 times per week showed no significant reduction in BPH risk [33]. Similarly, Hong et al. (2006) observed that men exercising 3–5 h per week had a lower risk of BPH, whereas those exercising nearly every day exhibited a 1.73-fold increased risk compared to those engaging in < 2 h per week [34]. On the other hand, Williams et al. (2008) identified a dose-response relationship, where greater running distances per week were associated with progressively lower BPH risk [36]. Nagakura et al. (2022) found a significant inverse correlation between exercise habits and BPH prevalence, with individuals engaging in regular sweaty exercise (≥ 30 min/session, ≥ 2 days/week) demonstrating a lower requirement for BPH medications [35].

Lee et al. (2014) found no significant association between exercise frequency or duration and BPH risk, noting that regular exercise (≥ 5 times per week) was associated with a higher risk [32], similar to the findings reported in Hong et al. (2006) [34]. The study also found that sedentary time (> 7 h per day) was associated with an increased risk of BPH [32]. Using a Mendelian randomization approach, Jia et al. (2024) found no evidence of a causal association between exercise intensity—including light, moderate, and vigorous activity—and BPH risk [37].

## PA and the Risk of BPH

Table 2 summarizes the key characteristics of the 14 studies investigating the association between PA and BPH risk, comprising five case-control studies [38–42], five cohort studies [13, 43–46], three cross-sectional studies [14, 32, 47] and one meta-analysis [26]. The meta-analysis conducted by Parsons and Kashefi (2008) included studies assessing both exercise and PA as interventions. The authors stratified the data according to light, moderate, and vigorous PA levels, which justified its inclusion in this Sect [26].

Seven of the 14 studies demonstrated an inverse correlation between PA and BPH [26, 40, 41, 43–45, 47], five studies did not establish a significant association between PA and BPH risk [13, 32, 38, 39, 42] and two studies reported mixed results [14, 46].

Among the seven studies demonstrating an inverse association between PA and BPH, the majority found that moderate-to-high intensity PA [26, 40, 41, 44, 47] provided greater protection than lower levels of PA [43]. The study of Platz et al. (1998) was the only study to conclude that low-to-moderate intensity PA was more effective than high-intensity PA [43], showing that men engaging in moderate activity had a 25% lower risk of BPH, while those in the highest-intensity category had a weaker association. The meta-analysis performed by Parsons and Kashefi (2008) found that moderate-to-high PA levels were equally protective, with both intensities significantly reducing BPH risk by 25% [26]. The studies of Meigs et al., 2001; Maso et al., 2006; Rohrmann et al., 2006 and Safarinejad, 2008 reported that high-intensity PA provided the greatest protection against BPH risk. Meigs et al. (2001) found that men in the highest PA quartile had a 50% lower risk of BPH compared to the least active group [44]. Similarly, Maso et al. (2006) observed a dose-dependent effect, where PA ≥ 5 h per week reduced BPH risk across different life stages, with the strongest effect in younger men [40]. Rohrmann et al. (2006) showed that higher PA scores, based on stair climbing and walking, were linked to lower LUTS prevalence, with the most active individuals experiencing the greatest benefit [41]. Safarinejad (2008) also reported a significant protective effect, with men expending ≥ 2000 kcal/day showing a 60% lower risk of BPH compared to those with ≤ 500 kcal/day [47]. Finally, Prezioso et al. (2001) found an overall protective effect of PA on prostate volume and LUTS severity but did not differentiate between PA intensity levels, making it unclear whether the benefit was driven by low, moderate, or high-intensity activity [45].

Age-related differences in the association between PA and the risk of BPH were also observed. Two studies [40, 43] demonstrated that in older men, low-to-moderate intensity PA was more protective against BPH than high-intensity. Conversely, in younger men, high-intensity PA conferred slightly greater protection compared to low-intensity PA [40, 43].

Among the studies reporting no association, PA was not significantly linked to BPH risk across various endpoints, including surgery, medical treatment, symptom severity, digital rectal examination, PSA screening, and prostate size [13, 32, 38, 39, 42]. Although Lee et al. (2014) observed no statistically significant difference in BPH risk between groups with high and low leisure-time PA, greater sedentary time was associated with increased prostate volume [32].

The studies reporting mixed results found no significant correlation between PA and prostate volume [14, 46] or BPH-related outcomes like reported or physician-diagnosed BPH, BPH surgery, finasteride use, and PSA levels [46]. However, higher PA levels were significantly associated with lower LUTS severity (*P* = 0.041) [14] and a 15% lower prevalence of severe nocturia in active men (≥ 1 h/week) [46], suggesting that PA may help alleviate LUTS without directly affecting prostate enlargement.

Overall, these findings underscore the methodological challenges in evaluating the association between exercise or PA and BPH risk and highlight the importance of interventional studies incorporating objective measurements and standardized endpoint definitions to determine whether exercise and/or PA confer protective effects against BPH.

## Limitations and Future Directions

The mixed results on the association between exercise or PA and BPH risk, highlight the challenges in conducting clinical research in this field. In addition to the substantial heterogeneity among studies — stemming from variations in study populations, analytical models (including covariates and the handling of different confounders), and exercise and PA assessment outcomes—the lack of a universally accepted definition of BPH exacerbates the complexities encountered both in epidemiological research and clinical practice. Several studies have evaluated BPH and LUTS as separate conditions, often utilizing questionnaires to examine the severity of LUTS without defining a specific threshold for BPH. This method results in LUTS being considered as an independent outcome. As a result, most research collects various metrics related to both BPH and LUTS, either examining each condition individually or combining them under a composite definition. These differences in approach can introduce variability in how outcomes are measured and interpreted, which hinders comparisons across studies and may lead to inconsistent findings regarding the relationship between exercise, PA and the risk of developing BPH and/or LUTS.

Case-control studies are susceptible to recall bias, as participants may inaccurately report past exercise or PA, leading to potential exposure misclassification. Additionally, undiagnosed BPH cases may be inadvertently included as controls, particularly in older populations where asymptomatic BPH is common, potentially skewing results. The retrospective nature of these studies introduces challenges in establishing a clear temporal relationship between exercise or PA and BPH risk, making it difficult to determine whether PA influenced BPH development or if other factors played a role. Cohort studies, although prospective, face issues like participant dropout and reliance on baseline exercise and PA assessments, which may not reflect changes over time. Cross-sectional studies, while useful for associations, cannot establish causality due to their single-timepoint design. These study types rely on self-reported data, which increases the risk of reporting bias, further confounding the interpretation of findings.

Future research should focus on randomized controlled trials, as they are essential for establishing causal links by rigorously controlling for confounding factors and randomly assigning participants to different interventions. To enhance research outcomes, future studies could benefit from incorporating objective and precise measures of exercise and PA, such as pedometers and accelerometers. Additionally, assessing attained levels of aerobic fitness, dose-response relationships, and exploring more precisely the underlying mechanisms will provide valuable insights into how exercise and PA affect the progression of BPH and LUTS, ultimately leading to the development of more effective prevention and intervention measures.

## Conclusions

Overall, observational studies suggest that moderate PA and exercise may protect against BPH and LUTS development, although causality remains uncertain and results are heterogeneous. Variations in exercise and PA assessment, BPH and LUTS definitions and outcomes, and imprecise study designs highlight the need for further research. Future randomized clinical trials are warranted to define causality, characterize dose-response relationships and explore the biological mechanisms underlying these associations to support evidence-based guidelines for clinical and public health recommendations.

###  Key References

- Jia F, Wei Z, Kong X, Mao Y, Yang Y. Causal Associations Between Lifestyle Habits and Risk of Benign Prostatic Hyperplasia: A Two-Sample Mendelian Randomization Study. Magaziner J, editor. J Gerontol Ser A. 2024 Jan 1;79(1):glad187. doi:10.1093/gerona/glad187
  - ◦This two-sample Mendelian randomization study demonstrates a robust, unidirectional causal association between longer sleep duration and reduced risk of benign prostatic hyperplasia. By minimizing confounding and reverse causality, it provides high-level evidence that adequate sleep is a modifiable protective factor, potentially mediated through testosterone regulation. Notably, sedentary behavior and varying levels of physical activity were not found to have a significant causal effect on BPH risk.
- Awedew AF, Han H, Abbasi B, Abbasi-Kangevari M, Ahmed MB, Almidani O, et al. The global, regional, and national burden of benign prostatic hyperplasia in 204 countries and territories from 2000 to 2019: a systematic analysis for the Global Burden of Disease Study 2019. Lancet Healthy Longev. 2022 Nov;3(11):e754–76. doi:10.1016/S2666-7568(22)00213-6.
  - ◦This Global Burden of Disease 2019 analysis quantifies the worldwide, regional, and national burden of benign prostatic hyperplasia, highlighting its rising prevalence and the importance of monitoring and planning for future health system strain. From our perspective, these data reinforce the urgent need to identify modifiable risk factors associated with BPH—particularly lifestyle determinants such as physical activity—to mitigate its growing public health impact.
- Wang YB, Yang L, Deng YQ, Yan SY, Luo LS, Chen P, et al. Causal relationship between obesity, lifestyle factors and risk of benign prostatic hyperplasia: a univariable and multivariable Mendelian randomization study. J Transl Med. 2022 Oct 29;20(1):495. doi:10.1186/s12967-022-03722-y.
  - ◦This Mendelian randomization study provides genetic evidence supporting a causal association between obesity, lifestyle-related factors, and BPH risk, strengthening the biological plausibility of metabolic contributions to disease pathogenesis. In the context of our manuscript, these findings reinforce the concept that modifiable lifestyle determinants—closely intertwined with physical activity and exercise patterns—play a mechanistic role in BPH development, thereby supporting preventive strategies targeting metabolic health.

This article does not contain any studies with human or animal subjects performed by any of the authors.

## Data Availability

No datasets were generated or analysed during the current study.
